# Exploratory study of ultraviolet B (UVB) radiation and age of onset of bipolar disorder

**DOI:** 10.1186/s40345-023-00303-w

**Published:** 2023-06-22

**Authors:** Michael Bauer, Tasha Glenn, Eric D. Achtyes, Martin Alda, Esen Agaoglu, Kürsat Altınbaş, Ole A. Andreassen, Elias Angelopoulos, Raffaella Ardau, Memduha Aydin, Yavuz Ayhan, Christopher Baethge, Rita Bauer, Bernhard T. Baune, Ceylan Balaban, Claudia Becerra-Palars, Aniruddh P. Behere, Prakash B. Behere, Habte Belete, Tilahun Belete, Gabriel Okawa Belizario, Frank Bellivier, Robert H. Belmaker, Francesco Benedetti, Michael Berk, Yuly Bersudsky, Şule Bicakci, Harriet Birabwa-Oketcho, Thomas D. Bjella, Conan Brady, Jorge Cabrera, Marco Cappucciati, Angela Marianne Paredes Castro, Wei-Ling Chen, Eric Y. W. Cheung, Silvia Chiesa, Marie Crowe, Alessandro Cuomo, Sara Dallaspezia, Maria Del Zompo, Pratikkumar Desai, Seetal Dodd, Bruno Etain, Andrea Fagiolini, Frederike T. Fellendorf, Ewa Ferensztajn-Rochowiak, Jess G. Fiedorowicz, Kostas N. Fountoulakis, Mark A. Frye, Pierre A. Geoffroy, Michael J. Gitlin, Ana Gonzalez-Pinto, John F. Gottlieb, Paul Grof, Bartholomeus C. M. Haarman, Hirohiko Harima, Mathias Hasse-Sousa, Chantal Henry, Lone Hoffding, Josselin Houenou, Massimiliano Imbesi, Erkki T. Isometsä, Maja Ivkovic, Sven Janno, Simon Johnsen, Flávio Kapczinski, Gregory N. Karakatsoulis, Mathias Kardell, Lars Vedel Kessing, Seong Jae Kim, Barbara König, Timur L. Kot, Michael Koval, Mauricio Kunz, Beny Lafer, Mikael Landén, Erik R. Larsen, Melanie Lenger, Rasmus W. Licht, Carlos Lopez-Jaramillo, Alan MacKenzie, Helle Østergaard Madsen, Simone Alberte Kongstad A. Madsen, Jayant Mahadevan, Agustine Mahardika, Mirko Manchia, Wendy Marsh, Monica Martinez-Cengotitabengoa, Julia Martini, Klaus Martiny, Yuki Mashima, Declan M. McLoughlin, Ybe Meesters, Ingrid Melle, Fátima Meza-Urzúa, Pavol Mikolas, Yee Ming Mok, Scott Monteith, Muthukumaran Moorthy, Gunnar Morken, Enrica Mosca, Anton A. Mozzhegorov, Rodrigo Munoz, Starlin V. Mythri, Fethi Nacef, Ravi K. Nadella, Takako Nakanotani, René Ernst Nielsen, Claire O’Donovan, Adel Omrani, Yamima Osher, Uta Ouali, Maja Pantovic-Stefanovic, Pornjira Pariwatcharakul, Joanne Petite, Johannes Petzold, Andrea Pfennig, Yolanda Pica Ruiz, Marco Pinna, Maurizio Pompili, Richard J. Porter, Danilo Quiroz, Francisco Diego Rabelo-da-Ponte, Raj Ramesar, Natalie Rasgon, Woraphat Ratta-apha, Michaela Ratzenhofer, Maria Redahan, M. S. Reddy, Andreas Reif, Eva Z. Reininghaus, Jenny Gringer Richards, Philipp Ritter, Janusz K. Rybakowski, Leela Sathyaputri, Angela M. Scippa, Christian Simhandl, Daniel Smith, José Smith, Paul W. Stackhouse, Dan J. Stein, Kellen Stilwell, Sergio Strejilevich, Kuan-Pin Su, Mythily Subramaniam, Ahmad Hatim Sulaiman, Kirsi Suominen, Andi J. Tanra, Yoshitaka Tatebayashi, Wen Lin Teh, Leonardo Tondo, Carla Torrent, Daniel Tuinstra, Takahito Uchida, Arne E. Vaaler, Eduard Vieta, Biju Viswanath, Maria Yoldi-Negrete, Oguz Kaan Yalcinkaya, Allan H. Young, Yosra Zgueb, Peter C. Whybrow

**Affiliations:** 1 Department of Psychiatry and Psychotherapy, University Hospital Carl Gustav Carus, Faculty of Medicine, Technische Universität Dresden, Dresden, Germany; 2 ChronoRecord Association, Fullerton, CA USA; 3 Division of Psychiatry and Behavioral Medicine, Michigan State University College of Human Medicine, Grand Rapids, MI USA; 4 Pine Rest Christian Mental Health Services, Grand Rapids, MI USA; 5 Department of Psychiatry, Dalhousie University, Halifax, NS Canada; 6 Department of Psychiatry, Hacettepe University Faculty of Medicine, Ankara, Turkey; 7 Department of Psychiatry, Selcuk University Faculty of Medicine, Mazhar Osman Mood Center, Konya, Turkey; 8 NORMENT Centre, Division of Mental Health and Addiction, Oslo University Hospital and Institute of Clinical Medicine, University of Oslo, Oslo, Norway; 9 Department of Psychiatry, National and Capodistrian University of Athens, Medical School, Eginition Hospital, Athens, Greece; 10 Section of Neurosciences and Clinical Pharmacology, Department of Biomedical Sciences, University of Cagliari, Sardinia, Italy; 11 Department of Psychiatry, Selcuk University Faculty of Medicine, Konya, Turkey; 12 Department of Psychiatry and Psychotherapy, University of Cologne Medical School, Cologne, Germany; 13 Department of Psychiatry, University of Münster, Münster, Germany; 14 Department of Psychiatry, Melbourne Medical School, The University of Melbourne, Melbourne, Australia; 15 The Florey Institute of Neuroscience and Mental Health, The University of Melbourne, Parkville, VIC Australia; 16 Department of Psychiatry, Psychosomatic Medicine and Psychotherapy, University Hospital Frankfurt, Johann Wolfgang Goethe-Universität Frankfurt Am Main, Frankfurt Am Main, Germany; 17 National Institute of Psychiatry “Ramón de la Fuente Muñiz”, Mexico City, Mexico; 18 Department of Pediatrics and Human Development, Michigan State University, Grand Rapids, MI USA; 19 Department of Psychiatry, Jawaharlal Nehru Medical College, Datta Meghe Institute of Medical Sciences (Deemed University), Wardha, India; 20 Department of Psychiatry, College of Medicine and Health Sciences, Bahir Dar University, Bahir Dar, Ethiopia; 21 Bipolar Disorder Research Program, Department of Psychiatry, University of São Paulo Medical School, São Paulo, Brazil; 22 Département de Psychiatrie et de Médecine Addictologique, Assistance Publique, Hôpitaux de Paris, INSERM UMR-S1144, Université Paris Cité, Fondation FondaMental, Paris, France; 23 Division of Psychiatry, Ben Gurion University of the Negev, Beer Sheva, Israel; 24 University Vita-Salute San Raffaele, Milan, Italy; 25 Psychiatry and Clinical Psychobiology, Division of Neuroscience, San Raffaele Scientific Institute, Milan, Italy; 26 IMPACT, The Institute for Mental and Physical Health and Clinical Translation, School of Medicine, Barwon Health, Deakin University, Geelong, Australia; 27 Orygen The National Centre of Excellence in Youth Mental Health, Centre for Youth Mental Health, Florey Institute for Neuroscience and Mental Health and the, Department of Psychiatry, The University of Melbourne, Melbourne, Australia; 28 Department of Psychiatry, Faculty of Health Sciences, Beer Sheva Mental Health Center, Ben Gurion University of the Negev, Beer Sheva, Israel; 29 Department of Psychiatry, Baskent University Faculty of Medicine, Ankara, Turkey; 30 Butabika Hospital, Kampala, Uganda; 31 Department of Psychiatry, Trinity College Dublin, St Patrick’s University Hospital, Dublin, Ireland; 32 Mood Disorders Clinic, Dr. Jose Horwitz Psychiatric Institute, Santiago de Chile, Chile; 33 Department of Mental Health and Substance Abuse, Piacenza, Italy; 34 Department of Psychiatry, Chiayi Branch, Taichung Veterans General Hospital, Chiayi, Taiwan; 35 Private Practice, Central, Hong Kong, China; 36 Department of Psychological Medicine, University of Otago, Christchurch, New Zealand; 37 Department of Molecular Medicine, University of Siena School of Medicine, Siena, Italy; 38 Department of Psychiatry, University of Melbourne, Parkville, VIC Australia; 39 Department of Psychiatry and Psychotherapeutic Medicine, Medical University Graz, Graz, Austria; 40 Department of Adult Psychiatry, Poznan University of Medical Sciences, Poznan, Poland; 41 Department of Psychiatry, School of Epidemiology and Public Health, University of Ottawa and The Ottawa Hospital, Ottawa, ON Canada; 42 3rd Department of Psychiatry, School of Medicine, Faculty of Health Sciences, Aristotle University of Thessaloniki, Thessaloniki, Greece; 43 Department of Psychiatry and Psychology, Mayo Clinic Depression Center, Mayo Clinic, Rochester, MN USA; 44 Département de Psychiatrie et d’addictologie, AP-HP, GHU Paris Nord, DMU Neurosciences, Hopital Bichat, Claude Bernard, 75018 Paris, France; 45 GHU Paris, Psychiatry and Neurosciences, 1 Rue Cabanis, 75014 Paris, France; 46 Université de Paris, NeuroDiderot, Inserm, FHU I2D2, 75019 Paris, France; 47 BIOARABA, Department of Psychiatry, University Hospital of Alava, University of the Basque Country, CIBERSAM, Vitoria, Spain; 48 Department of Psychiatry, Feinberg School of Medicine, Northwestern University, Chicago, IL USA; 49 Mood Disorders Center of Ottawa and the Department of Psychiatry, University of Toronto, Toronto, Canada; 50 Department of Psychiatry, University Medical Center Groningen, University of Groningen, Groningen, The Netherlands; 51 Department of Psychiatry, Tokyo Metropolitan Matsuzawa Hospital, Setagaya, Tokyo, Japan; 52 Department of Psychiatry, Universidade Federal do Rio Grande do Sul, Porto Alegre, Brazil; 53 Programa de Pós-Graduação em Psicologia, Departamento de Psicologia do Desenvolvimento e da Personalidade, Instituto de Psicologia, Universidade Federal do Rio Grande do Sul, Porto Alegre, Brazil; 54 Department of Psychiatry, GHU Paris Psychiatrie and Neurosciences, Université de Paris, F-75014, F-75006 Paris, France; 55 Department of Clinical Research, University of Southern Denmark, Odense, Denmark; 56 Université Paris Est Créteil, INSERM, IMRB, Translational Neuropsychiatry, APHP, Mondor Univ Hospitals, Fondation FondaMental, F-94010 Créteil, France; 57 Université Paris Saclay, CEA, Neurospin, F-91191 Gif-Sur-Yvette, France; 58 Department of Psychiatry, University of Helsinki and Helsinki University Hospital, Helsinki, Finland; 59 National Institute for Health and Welfare, Helsinki, Finland; 60 Clinic for Psychiatry, University Clinical Center of Serbia, Belgrade, Serbia; 61 Department of Psychiatry, University of Tartu, Tartu, Estonia; 62 Unit for Psychiatric Research, Aalborg University Hospital, Aalborg, Denmark; 63 Department of Psychiatry and Neurochemistry, Institute of Neuroscience and Physiology, The Sahlgrenska Academy, University of Gothenburg, Gothenburg, Sweden; 64 Copenhagen Affective Disorder Research Center (CADIC), Psychiatric Center Copenhagen, Copenhagen, Denmark; 65 Department of Psychiatry, Chosun University School of Medicine, Gwangju, Republic of Korea; 66 BIPOLAR Zentrum Wiener Neustadt, Wiener Neustadt, Austria; 67 Khanty-Mansiysk Clinical Psychoneurological Hospital, Khanty-Mansiysk, Russia; 68 Neuroscience Program, Michigan State University, East Lansing, MI USA; 69 Department of Medical Epidemiology and Biostatistics, Karolinska Institutet, Stockholm, Sweden; 70 Mental Health Department Odense, University Clinic and Department of Regional Health Research, University of Southern Denmark, Esbjerg, Denmark; 71 Psychiatry, Aalborg University Hospital, Aalborg, Denmark; 72 Department of Clinical Medicine, Aalborg University, Aalborg, Denmark; 73 Mood Disorders Program, Hospital Universitario San Vicente Fundación, Research Group in Psychiatry, Department of Psychiatry, Faculty of Medicine, Universidad de Antioquia, Medellín, Colombia; 74 Forensic Psychiatry, University of Glasgow, NHS Greater Glasgow and Clyde, Glasgow, UK; 75 Psychiatric Centre Copenhagen, Copenhagen University Hospitals, Copenhagen, Denmark; 76 Department of Psychiatry, National Institute of Mental Health and Neuro Sciences (NIMHANS), Bengaluru, India; 77 Department of Psychiatry, Faculty of Medicine, Mataram University, Mataram, Indonesia; 78 Department of Pharmacology, Dalhousie University, Halifax, NS Canada; 79 Section of Psychiatry, Department of Medical Science and Public Health, University of Cagliari, Cagliari, Italy; 80 Unit of Clinical Psychiatry, University Hospital Agency of Cagliari, Cagliari, Italy; 81 Department of Psychiatry, University of Massachusetts Medical School, Worcester, MA USA; 82 Osakidetza, Basque Health Service, BioAraba Health Research Institute, University of the Basque Country, Bilbao, Spain; 83 The Psychology Clinic of East Anglia, Norwich, UK; 84 Department of Neuropsychiatry, Keio University School of Medicine, Tokyo, Japan; 85 Department of Psychiatry and Trinity College Institute of Neuroscience, Trinity College Dublin, St Patrick’s University Hospital, Dublin, Ireland; 86 Department of Child and Adolescent Psychiatry Und Psychotherapy, SHG Klinikum, Idar-Oberstein, Germany; 87 Department of Mood and Anxiety Disorders, Institute of Mental Health, Singapore City, Singapore; 88 Michigan State University College of Human Medicine, Traverse City Campus, Traverse City, MI USA; 89 Department of Mental Health, Norwegian University of Science and Technology, NTNU, Trondheim, Norway; 90 Department of Psychiatry, St Olavs’ University Hospital, Trondheim, Norway; 91 Soviet Psychoneurological Hospital, Urai, Russia; 92 Department of Psychiatry, University of California San Diego, San Diego, CA USA; 93 Makunda Christian Leprosy and General Hospital, Bazaricherra, Assam 788727 India; 94 Razi Hospital, Faculty of Medicine, University of Tunis-El Manar, Tunis, Tunisia; 95 Tokyo Metropolitan Hiroo Hospital, 2-34-10 Ebisu, Shibuya-Ku, Tokyo, 150-0013 Japan; 96 Tunisian Bipolar Forum, Érable Médical Cabinet 324, Lac 2, Tunis, Tunisia; 97 Department of Psychiatry, Faculty of Medicine Siriraj Hospital, Mahidol University, Bangkok, Thailand; 98 Hospital “Ángeles del Pedregal”, Mexico City, Mexico; 99 Lucio Bini Mood Disorder Center, Cagliari, Italy; 100 Department of Neurosciences, Mental Health and Sensory Organs, Sant’Andrea Hospital, Sapienza University of Rome, Rome, Italy; 101 Deparment of Psychiatry, Diego Portales University, Santiago de Chile, Chile; 102 School of Pharmacy and Biomedical Sciences, University of Central Lancashire, Preston, Lancashire UK; 103 SA MRC Genomic and Precision Medicine Research Unit, Division of Human Genetics, Department of Pathology, Institute of Infectious Diseases and Molecular, Medicine, University of Cape Town, Cape Town, South Africa; 104 Department of Psychiatry and Behavioral Sciences, Stanford School of Medicine, Palo Alto, CA USA; 105 Asha Bipolar Clinic, Asha Hospital, Hyderabad, Telangana India; 106 Departments of Psychiatry, Epidemiology, and Internal Medicine, Iowa Neuroscience Institute, The University of Iowa, Iowa City, IA USA; 107 Department of Neuroscience and Mental Health, Federal University of Bahia, Salvador, Brazil; 108 Bipolar Zentrum Wiener Neustadt, Sigmund Freud Privat Universität, Vienna, Austria; 109 Centre for Clinical Brain Sciences, University of Edinburgh, Edinburgh, Scotland, UK; 110 AREA, Assistance and Research in Affective Disorders, Buenos Aires, Argentina; 111 Science Directorate/Climate Science Branch, NASA Langley Research Center, Hampton, VA USA; 112 Department of Psychiatry, MRC Unit on Risk and Resilience in Mental Disorders, University of Cape Town, Cape Town, South Africa; 113 College of Medicine, China Medical University (CMU), Taichung, Taiwan; 114 An-Nan Hospital, China Medical University, Tainan, Taiwan; 115 Research Division, Institute of Mental Health, Singapore, Singapore; 116 Department of Psychological Medicine, Faculty of Medicine, University of Malaya, Kuala Lumpur, Malaysia; 117 Department of Social Services and Health Care, Psychiatry, City of Helsinki, Helsinki, Finland; 118 Department of Psychiatry, Faculty of Medicine, Hasanuddin University, Makassar, Indonesia; 119 McLean Hospital-Harvard Medical School, Boston, MA USA; 120 Mood Disorder Lucio Bini Centers, Cagliari e Rome, Italy; 121 Clinical Institute of Neuroscience, Hospital Clinic, University of Barcelona, IDIBAPS, CIBERSAM, Barcelona, Catalonia Spain; 122 Melbourne Neuropsychiatry Centre, Department of Psychiatry, The University of Melbourne, Melbourne, Australia; 123 Subdirección de Investigaciones Clínicas, Instituto Nacional de Psiquiatría Ramón de la Fuente Muñíz, Mexico City, Mexico; 124 Department of Psychological Medicine, Institute of Psychiatry, Psychology and Neuroscience, King’s College London, London, UK; 125 Department of Psychiatry and Biobehavioral Sciences, Semel Institute for Neuroscience and Human Behavior, University of California Los Angeles (UCLA), Los Angeles, CA USA

## Abstract

**Background:**

Sunlight contains ultraviolet B (UVB) radiation that triggers the production of vitamin D by skin. Vitamin D has widespread effects on brain function in both developing and adult brains. However, many people live at latitudes (about > 40 N or S) that do not receive enough UVB in winter to produce vitamin D. This exploratory study investigated the association between the age of onset of bipolar I disorder and the threshold for UVB sufficient for vitamin D production in a large global sample.

**Methods:**

Data for 6972 patients with bipolar I disorder were obtained at 75 collection sites in 41 countries in both hemispheres. The best model to assess the relation between the threshold for UVB sufficient for vitamin D production and age of onset included 1 or more months below the threshold, family history of mood disorders, and birth cohort. All coefficients estimated at P ≤ 0.001.

**Results:**

The 6972 patients had an onset in 582 locations in 70 countries, with a mean age of onset of 25.6 years. Of the onset locations, 34.0% had at least 1 month below the threshold for UVB sufficient for vitamin D production. The age of onset at locations with 1 or more months of less than or equal to the threshold for UVB was 1.66 years younger.

**Conclusion:**

UVB and vitamin D may have an important influence on the development of bipolar disorder. Study limitations included a lack of data on patient vitamin D levels, lifestyles, or supplement use. More study of the impacts of UVB and vitamin D in bipolar disorder is needed to evaluate this supposition.

## Background

The sunlight that penetrates the atmosphere and reaches the Earth’s surface has profound effects on human physiology and behavior, and is fundamental to human health (Wirz-Justice 2021). Daylight is the most powerful signal to entrain the human circadian system to the 24 h rotation of the Earth (Foster 2021; Roenneberg 2007). Daylight contains ultraviolet B radiation (UVB) that is absorbed by skin, triggers production of vitamin D, and is the major source of vitamin D for both children and adults (Holick 2017). Some of the many aspects of human health affected by daylight include sleep, mood, alertness, cognition, bone health, calcium homeostasis, neuroendocrine and cardiovascular regulation, and eyesight (Wirz-Justice 2021; Holick 2004; Paul 2019; LeGates 2014; Blume 2019; Crnko 2019; Lagreze 2017).

In prior studies, we analyzed the impact of solar insolation (incoming solar radiation) on several aspects of bipolar I disorder. Solar insolation is defined as the total amount of electromagnetic energy from the Sun striking a surface area of the Earth, and includes all wavelengths of visible and invisible light (NASA 2021). An inverse relation was found between the maximum monthly increase in solar insolation in springtime and the age of onset of bipolar I disorder (Bauer 2017). Due to the frequent symptoms of circadian disruption in patients with bipolar disorder, our studies of solar insolation focused the discussion on visible light and circadian entrainment (Bellivier 2015; Gonzalez 2014; Takaesu 2018).

The purpose of this exploratory analysis was to investigate the association between UVB and the age of onset of bipolar I disorder using a large, global sample. Recent findings emphasize the broad range of non-skeletal vitamin D functions including actions on the developing and adult brain and the association of vitamin D deficiency with neurological and psychiatric disorders (Cuomo 2019; Hoilick 2004; Mayne 2019; Cui 2021; Bailon 2012; Berk 2009; Patrick 2015; Eyles 2020). Although UVB is approximately the same proportion of the total broadband solar insolation at all locations, many people live at latitudes that do not receive enough UVB during winter months to produce vitamin D from skin absorption (Webb 1988; Wacker 2013). The association of the age of onset of bipolar disorder with UVB is of particular importance given the high global rate of vitamin D deficiency (Holick 2017; Palacios 2014), and relevance of the age of onset to the outcome in bipolar disorder (Joslyn 2016; Menculini 2022).

## Methods

All patients included in the study had a diagnosis of bipolar disorder made by a psychiatrist according to DSM-IV or DSM-5 criteria. The researchers were from university medical centers and specialty clinics, as well as individual practitioners. Data were collected retrospectively between 2010 and 2016 and 2019–2021, by patient questioning, record review or both. Details about the methodology for data collection were previously published (Bauer 2012; Bauer 2017; Bauer 2022). Study approval, including for data collection, was obtained according to local requirements, using local institutional review boards.

### Data collection sites

Data were obtained at 75 data collection sites located in 41 countries in both hemispheres. The data collection sites in the northern hemisphere were in Austria: Graz, Wiener Neustadt; Canada: Calgary, Halifax, Ottawa; China: Hong Kong; Colombia: Medellín; Denmark: Aalborg, Aarhus, Copenhagen; Ethiopia: Barhir Dar; Estonia: Tartu; Finland: Helsinki; France: Paris (2 sites);Germany: Dresden, Frankfurt, Würzburg; Greece: Athens, Thessaloniki (2 sites); India: Bengaluru, Hyderabad, Wardha; Ireland: Dublin; Israel: Beer Sheva; Italy: Cagliari, Sardinia (2 sites), Milan, Piacenza, Rome, Siena; Japan: Tokyo (3 sites); Malaysia: Kuala Lumpur; Mexico: Mexico City; Netherlands: Groningen; Norway: Oslo, Trondheim; Poland: Poznan; Russia: Khanti-Mansiysk; Serbia: Belgrade; Singapore; South Korea: Jincheon; Spain: Barcelona, Vitoria; Sweden; Gothenburg; Stockholm; Taiwan: Taichung; Thailand: Bangkok; Turkey: Ankara; Konya; Tunisia: Tunis; Uganda: Kampala; UK: Glasgow; and USA: Grand Rapids, MI, Iowa City, IA, Kansas City, KS, Los Angeles, CA, Palo Alto, CA, Rochester, MN, San Diego, CA, and Worcester, MA. The collection sites in the southern hemisphere were in Australia: Adelaide, Melbourne/Geelong; Argentina: Buenos Aires; Brazil: Porto Alegre, Salvador, São Paulo; Chile: Santiago (2 sites); Indonesia: Mataram; New Zealand: Christchurch; and South Africa: Cape Town.

### Data variables

The data collected for each patient included sex, age of onset, onset location, family history of mood disorders, polarity of first episode, history of psychosis, episode course, history of alcohol and substance abuse, and history of suicide attempts. Four birth cohort groups were used: born before 1940, between 1940 and 1959, between 1960 and 1979, and after 1979.

All the patient actual onset locations were grouped into reference onset locations, which represent all the onset locations within a 1 × 1 degree grid of latitude and longitude. The reference onset location was used to obtain the UVB data for each patient and used in the analysis.

### UVB

The surface UVB (280–315 nm) data are estimated by the NASA CERES ((Clouds and Earth’s Radiant Energy System), Wielicki 1996; Su 2005) and were downloaded from the NASA POWER database, based on 20-year Meteorological and Solar Monthly & Annual Climatologies (January 2001—December 2020), and accessed via the POWER Climatology API, Version: v2.2.22 (NASA 2022). For each reference onset location, the monthly average UVB expressed in watts/square meter (W/m^2^), and the daily average daylight hours for each month were obtained. For consistency with prior research, the average monthly W/m^2^ values for UVB for each reference site were converted to the average total daily kilojoule/square meter as:

$${\text{kJ}}/{\text{m}}^{{2}} /{\text{day }} = {\text{ W}}/{\text{m}}^{{2}} *{ 36}00/{1}000 \, *{\text{ daylight hours}}$$

for each month.

**Fig. 1:**
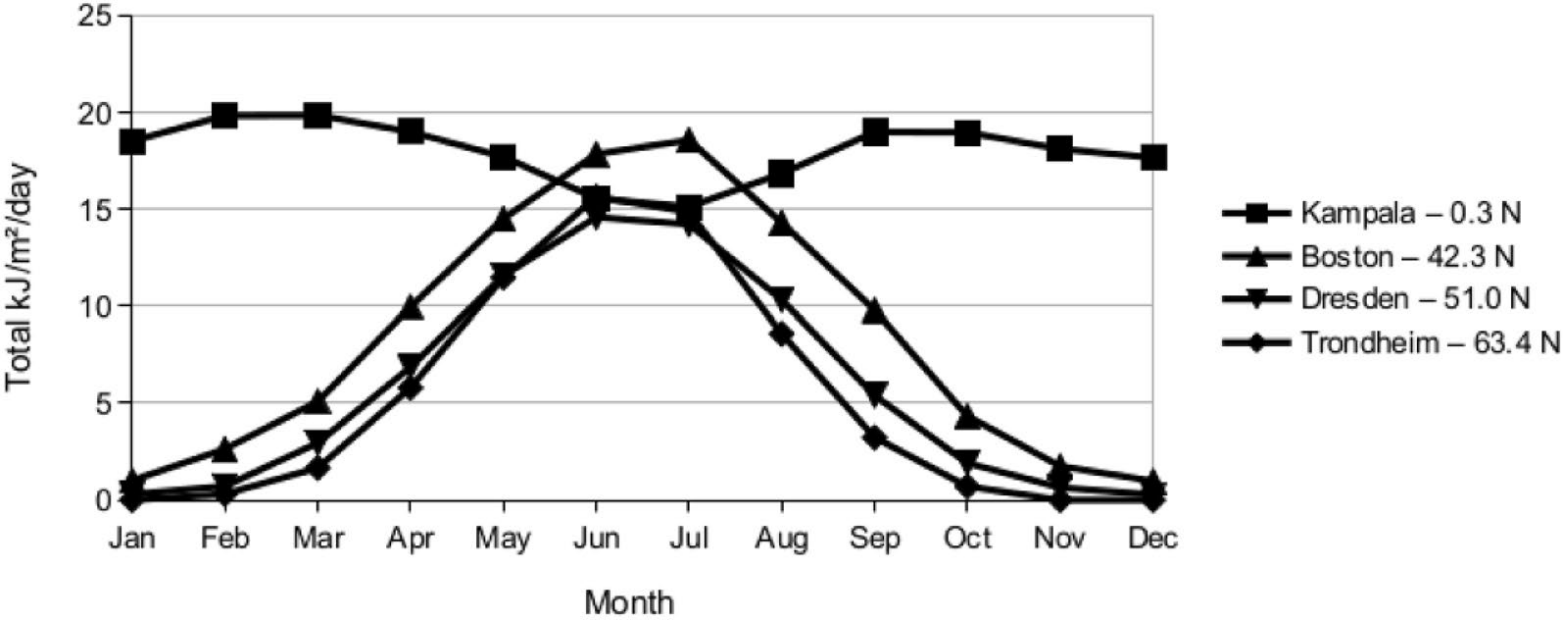
Total UVB kJ/m^2^/day by Month for Selected Reference Locations

The UVB received at the Earth’s surface varies greatly by geographical location. See Fig. 1. UVB transmission through the atmosphere is greatly reduced by clouds, ozone and heavy air pollution (NASA 2022; Su 2005). For locations at the same latitude with similar cloud patterns, increasing elevation will increase surface UVB.

Above approximately 40° latitude N or S, there is insufficient UVB for vitamin D synthesis in winter (November through February in the northern hemisphere) (Webb 1988; Holick 2004). This study analyzed the relation between the threshold for UVB sufficient for vitamin D production in skin and the age of onset of bipolar disorder. Several researchers have estimated thresholds from 0.7 to 1.0 kJ/m^2^/day UVB (McKenzie 2009; O’Neill 2016). This analysis used a threshold of 0.75 kJ/m^2^/day UVB.

### Statistics

The generalized estimating equations (GEE) statistical technique was used to accommodate the correlated data, and unbalanced number of patients within each reference onset location. The GEE model uses a marginal or population-averaged approach, to estimate the effect across the entire population rather than within a cluster (Zeger 1986). The dependent variable was the age of onset. An exchangeable correlation matrix was selected, which is appropriate for a large number of clusters including many with a single observation (Stedman 2008). Sidak’s adjustment for multiple comparisons was used to make pair-wise comparisons between the birth-cohorts. A significance level of 0.001 was used for all evaluations to reduce the chance of type I error. The corrected quasi-likelihood independence model criterion was used to assist with model fitting (Pan 2001). SPSS version 28.0.0.0 was used for all analyses.

## Results

Data for 11,063 patients with bipolar disorder were obtained from the 75 collection sites, including 8080 patients with a diagnosis of bipolar I disorder. Of those with bipolar I disorder, 6972 patients had all variables in the best model. The demographic characteristics of the 6972 patients with bipolar I disorder are shown in Table 1. The mean age of onset for the 6972 patients was 25.6 years, shown distributed by latitude range in Table 2. The 6972 patients had an onset in 582 onset locations in 70 countries. There was a mean of 12 patients at each onset location, with 4% of the 582 locations having only one patient. Of the 6972 patients, 1293 (18.5%) had an onset in the southern hemisphere, and 1598 (22.9%) had an onset in the tropics.

**Table 1:** Demographics of Bipolar I patients^a^ (N = 6972)

Parameter	Value	N	%
Gender			
	Female	4054	58.3
	Male	2894	41.7
First Episode			
	Manic/Hypomanic	3384	50.2
	Depressed	3358	49.8
Family History of Mood Disorder			
	No	3328	47.7
	Yes	3644	52.3
History of Alcohol or Substance Abuse			
	No	3492	69.5
	Yes	1531	30.5
History of psychosis			
	No	1986	35.4
	Yes	3622	64.6
Comorbid Anxiety/Panic/OCD			
	No	3854	77.4
	Yes	1123	22.6
Cohort Age Group			
	DOB after 1979	1732	24.8
	DOB 1979–1960	3234	46.4
	DOB 1959–1940	1738	24.9
	DOB before 1940	268	3.8
Onset Hemisphere			
	Northern	5679	81.5
	Southern	1293	18.5
Parameter		Mean	SD
Age of Onset		25.6	10.4

^a^Missing values excluded

**Table 2:** Mean Age of Onset by Latitude Range (N = 6972)

Latitude Range North + South	Mean Age of Onset	N	Standard Deviation
0–9	26.6	511	9.97
10–19	24.3	797	9.49
20–29	24.8	375	11.39
30–39	25.5	2023	10.38
40–49	26.6	2368	10.59
50–59	24.4	682	10.16
60–69	22.7	216	11.29
Total	25.6	6972	10.43

The best fitting model estimated the age of onset using an intercept, 1 or more months of less than 0.75 mean monthly kJ/m^**2**^/day of UVB at the patient onset location, family history of mood disorders and patient birth cohort. All estimated coefficients were significant at the P < 0.001 level. The age of onset for patients at an onset location with at least 1 month < 0.75 mean monthly kJ/m^2^/day of UVB was 1.66 (99% CI [-2.614, -0.712]) years younger than for patients at an onset location elsewhere as shown in Table 3**.**

**Table 3:** Estimated parameters explaining age of onset for patients with bipolar I disorder below a threshold of mean monthly kJ/m^2^/day of 0.75 UVB light for 1 or more months during the year (N = 6972)

			99% Confidence Interval		Coefficient Significance	
Parameters	Coefficient estimate (β)	Standard Error	Lower	Upper	Wald Chi-squared	P
Intercept	40.300	1.0867	38.107	42.429	1375.305	< 0.001
Family history of mood disorders	− 1.914	0.2316	− 2.368	− 1.460	68.309	< 0.001
Cohort age groups						
DOB after 1979	− 19.768	1.0344	− 21.796	− 17.741	365.219	< 0.001
DOB 1979–1960	− 13.575	1.0509	− 15.635	− 11.516	166.865	< 0.001
DOB 1959–1940	− 7.509	1.0339	− 9.536	− 5.483	52.750	< 0.001
DOB before 1940	0					
UVB kJ/m^2^/day < 0.75 for 1 or more months	− 1.663	0.4853	− 2.614	− 0.712	11.739	< 0.001

Dependent variable: Age of onset (years). Model: intercept, family history of mood disorders (Y/N), cohort age groups, UVB kJ/m^2^/day < 0.75 for 1 or more months (Y/N). All Sidak pairwise comparisons between family history of mood disorders and cohort age groups were significant at the < 0.001 level

Of the 582 onset locations, 198 (34.0%) had at least 1 month of less than 0.75 mean monthly kJ/m^**2**^/day of UVB. All of these onset locations were at latitudes of 40 degrees or greater N or S, and included 2247 (32.2%) of patients.

## Discussion

An association between UVB and the age of onset of bipolar disorder was observed. Patients at locations with 1 or more months of less than the threshold for UVB sufficient for vitamin D production had an onset that was 1.66 years younger. However, there are major limitations to this exploratory study. There is no data on patient vitamin D levels, lifestyle, sun exposure, sunscreen use or if taking vitamin D supplements. There is no data on dietary habits, although lower vitamin D levels were reported in vegetarians (Crowe 2011), or on skin pigmentation which effects absorption of UV radiation (Jablonski 2010). There is no data on whether patients take medications that interact with vitamin D such as many anti-epileptic drugs (Wakeman 2021; Fan 2016). There is no data on country vitamin D fortification. Yet, despite these limitations, an association between UVB and the age of onset of bipolar disorder was seen. This suggests that the role of UVB and vitamin D in bipolar disorder needs to be studied.

Vitamin D deficiency is frequently present in patients with psychiatric disorders. Many studies have reported vitamin D deficiency in patients with schizophrenia and major depressive disorder (MDD), with some opposite findings in MDD (Cui 2021; Valipour 2014; Bivona 2019). There are fewer studies of patients with bipolar disorder, but vitamin D status in these patients was similar to that of patients with other psychiatric disorders (Cereda 2021). The frequent medical comorbidity in patients with bipolar disorder may lead to poor eating habits, and limit exercise and sunlight exposure, which may also contribute to findings of vitamin D deficiency (Eyles 2020). Additionally, vitamin D deficiency and insufficiency is very common in international studies of people admitted for inpatient psychiatric treatment (Seiler 2022).

Vitamin D is a neurosteroid that has multiple roles in the brain throughout life. Vitamin D is involved in regulating brain development, maintaining function in the adult brain, and protecting the aged brain (Cui 2021; Eyles 2020; Groves 2014). Vitamin D acts on the brain by both genomic and non-genomic pathways. The genomic pathway involves vitamin D receptors that are found throughout most regions of the brain (Cui 2021; Eyles 2020; Groves 2014). The actions of vitamin D within the brain influence neurotransmission, neuroprotection, synaptic plasticity, immunomodulation, and calcium signaling. This includes involvement of vitamin D in the release of neurotransmitters including dopamine, gamma-aminobutyric acid (GABA), and serotonin, and neuroprotective effects that suppress oxidative stress and inhibit inflammation (Cui 2021; Eyles 2020; Groves 2014). The role of vitamin D in the development and severity of psychiatric disorders is an area of active research, including for bipolar disorder (Berridge 2017; Berridge 2015; Patrick 2015; Eghtedarian 2022; Eyles 2013).

## Other Limitations

Details about vitamin D production, and mechanisms of action in the brain were out of scope. Issues related to vitamin D assay methods, and differences between international guidelines for thresholds and supplementation were not discussed (Guistina 2020; Bouillon 2017). Changing needs for vitamin D across the lifespan, and strategies to address global vitamin D deficiency were not discussed (Bouillon 2017; Mendes 2020). UVB-related pathologies related to excessive exposure including skin cancers and ocular diseases were not discussed (Gies 2018). The potential use of vitamin D supplements as a treatment for bipolar disorder was out of scope (Marsh 2017). The surface UVB values were estimated from available satellite data and may differ slightly from direct surface UVB measurements (Su 2005). The global procedures implemented to prevent depletion of stratospheric ozone and resultant decreases in UVB were not included (Barnes 2019; NASA 2021).

## Conclusion

UVB is fundamental to the development of vitamin D, which is widely involved in the regulation of brain activities. In this large global study, patients at locations where the available UVB was below the threshold required for vitamin D production for at least 1 month had a younger age of onset of bipolar I disorder. UVB and vitamin D may have an important influence on the development of bipolar disorder. Further investigation of the role of UVB exposure and vitamin D in bipolar disorder is needed to evaluate this supposition.

## Data Availability

The data will not be shared or made publicly available.
